# Mental health and well-being of Children in France : Enabee the 1st nationwide study

**DOI:** 10.1192/j.eurpsy.2023.998

**Published:** 2023-07-19

**Authors:** S. Monnier-Besnard, A.-L. Perrine, L. Seconda, Y. Motreff, M. Marillier, V. Decio, D. Pognon, C. Verdot, M. El Haddad, A. Saoudi, V. Kovess, J.-B. Richard, N. Regnault

**Affiliations:** 1 Direction des Maladies Non Transmissibles et Traumatismes; 2 Direction Appui, Traitement et Analyses de données, Santé publique France, Saint-Maurice; 3 Université de Paris, Paris, France

## Abstract

**Introduction:**

The Covid 19 pandemic has worsened mental health of teenagers and young adults in particular and highlighted the lack of data for children aged 3 -11 years living in France. To fill this gap, Santé publique France, the national public health agency set up the first nationwide study, Enabee, in 2022.

**Objectives:**

Enabee (National study on Children wellbeing) aims at monitoring wellbeing and most frequent mental health disorders of children and at understanding associated factors, gathering information from children, parents and teachers. First analyses will be focused on children’ and teachers’ point of view.

**Methods:**

Enabee is a nationwide cross sectional study. Elementary and nursery schools were randomly selected in Metropolitan France. Then a maximum of four classes were randomly selected in each school. Elementary school children (from 6 to 11 years old) gave their own assessment of wellbeing and mental health using the following self-administrated questionnaires on tablets: the Kindl and the Dominique Interactive. To get a comprehensive evaluation, parents and teachers also filled the web-administrated Strengths and Difficulties Questionnaire for each child. The parents’ questionnaire also included questions on child’s life habits and global health, parenting attitudes, parent’s mental health, covid 19, major life events and household social situation. A pilot study was launched in January to assess the feasibility and the acceptance before implementing the study at a nationwide level. Key stakeholders of education, family and health participated at the setting up of the study.

**Results:**

706 schools were selected and 399 participated (participation rate 57%). Data were collected from May 2^nd^ to July 31^st^ 2022. In those schools, 1357 classes participated, representing 29 414 children. We collected 15 206 questionnaires filled by children of elementary schools and 21 016 questionnaires filled by teachers for children in nursery and elementary schools. Analysis are ongoing. By March 2023, we will produce weighted estimates of prevalence of children internal and externals disorders based on the children self-assessment and the teachers’ assessment respectively and different dimensions of wellbeing. Prevalences will be presented by sex, age and school levels.

**Conclusions:**

Enabee will provide a representative picture of French children wellbeing and mental health and protective and risks factors. This milestone is essential to guide national policies and build dedicated actions for children in order to promote and improve their wellbeing and mental health. Beyond this edition, Enabee is the first step of a long term monitoring system that will provide regularly updated data and will be completed by ancillary and ad hoc studies.

**Disclosure of Interest:**

None Declared

